# Understanding the role of magnetic (Fe_3_O_4_) nanoparticle to mitigate cadmium stress in radish (*Raphanus sativus* L.)

**DOI:** 10.1186/s40529-024-00420-4

**Published:** 2024-07-12

**Authors:** Amina Aslam, Zahra Noreen, Madiha Rashid, Muhammad Aslam, Tanveer Hussain, Afifa Younas, Sajid Fiaz, Kotb A. Attia, Arif Ahmed Mohammed

**Affiliations:** 1 Department of Botany, Division of Science and Technology, University of Education, Lahore, Pakistan; 2 Department of Horticulture, PMAS Arid Agriculture University Rawalpindi, Rawalpindi, Pakistan; 3 Lahore College for Women University Lahore, Jinnah Town, Lahore, Punjab, 44444 Pakistan; 4 Department of Plant Breeding and Genetics, The University of Haripur, Haripur, 22620 KP Pakistan; 5 School of Life Sciences, Henan Institute of Science and Technology, Xinxiang City, Henan Province China; 6 Department of Biochemistry, College of Science, King Saud University, P.O. Box 2455, Riyadh, 11451 Saudi Arabia

**Keywords:** Nanoparticles, MDA content, Soluble protein contents, Ion concentrations, Membrane permeability

## Abstract

Heavy metals stress particularly cadmium contamination is hotspot among researchers and considered highly destructive for both plants and human health. Iron is examined as most crucial element for plant development, but it is available in inadequate amount because they are present in insoluble Fe^3+^ form in soil. Fe_3_O_4_ have been recently found as growth promoting factor in plants. To understand, a sand pot experiment was conducted in completely randomized design (control, cadmium, 20 mg/L Fe_3_O_4_ nanoparticles,40 mg/L Fe_3_O_4_ nanoparticles, 20 mg/L Fe_3_O_4_ nanoparticles + cadmium, 40 mg/L Fe_3_O_4_ nanoparticles + cadmium) to study the mitigating role of Fe_3_O_4_ nanoparticles on cadmium stress in three *Raphanus sativus c*ultivars namely i.e., MOL SANO, MOL HOL PARI, MOL DAQ WAL. The plant growth, physiological and biochemical parameters i.e.,shoot length, shoot fresh weight, shoot dry weight, root length, root fresh and dry weight, MDA content, soluble protein contents, APX, CAT, POD activities and ion concentrations, membrane permeability, chlorophyll a, chlorophyll b and anthocyanin content, respectively were studied. The results displayed that cadmium stress remarkably reduces all growth, physiological and biochemical parameters for allcultivars under investigation. However, Fe_3_O_4_ nanoparticles mitigated the adverse effect of cadmium by improving growth, biochemical and physiological attributes in all radish cultivars. While, 20 mg/L Fe_3_O_4_ nanoparticles have been proved to be more useful against cadmium stress. The outcome of present investigation displayed that Fe_3_O_4_ nanoparticles can be utilized for mitigating heavy metal stress.

## Introduction

Radish (*Raphanus sativus* L.) is a root vegetable belonging to family *cruciferae* and traditionally considered an economically important vegetable, worldwide. It can be cultivated annually or biennially, which varies throughout the world. It can also be utilized as a potential medicinal plant fodder and green manure (Zaki et al. 2012). Radish (*Raphanus sativus* L.) is a winter vegetable crop that was cultivated on 10,153 hectares, in Pakistan, with the production of 16,8257 tons during year 2013–2014. The world production of radish is estimated to be about 7 million tons per year, representing roughly 2% of all vegetables (Kopta and Pokluda 2013). Radish can be used or served cooked or in raw form and can also be preserved for later use. Some varieties can be preserved with salt or in dried form, while some varieties can only be used fresh. The leaves and roots of radishes contain a large quantity of vitamin C. The radish has anti-cancer properties and has been used for the treatment of liver disorders for a long time (Pirdosti et al. 2019).

Radish (*Raphanus sativus* L.) is a root vegetable belonging to family *cruciferae* and traditionally considered an economically important vegetable, worldwide. It can be cultivated annually or biennially, which varies throughout the world. It can also be utilized as a potential medicinal plant fodder and green manure (Zaki et al. 2012). Radish (*Raphanus sativus* L.) is a winter vegetable crop that was cultivated on 10,153 hectares, in Pakistan, with the production of 16,8257 tons during year 2013–2014. The world production of radish is estimated to be about 7 million tons per year, representing roughly 2% of all vegetables (Kopta and Pokluda 2013). Radish can be used or served cooked or in raw form and can also be preserved for later use. Some varieties can be preserved with salt or in dried form, while some varieties can only be used fresh. The leaves and roots of radishes contain a large quantity of vitamin C. The radish has anti-cancer properties and has been used for the treatment of liver disorders for a long time (Pirdosti et al. 2019).

Cadmium is a toxic heavy metal, which can severely damage plant health and equally harmful for human health. Heavy metals present in soil can easily be absorbed through roots and reached to different parts of plants (Gill et al. 2013), and moved to human bodies through the food chain (Kao 2014). Cadmium can affect the plants’ growth processes by causing severe changes in enzyme activities, mineral nutrition transpiration, biosynthesis of nucleic acids and chlorophyll, reactive oxygen species (ROS) production etc. These changes results in damages to plants like necrosis, chlorosis, browning of the root, and even death (Asgher et al. 2015). The toxicity of heavy metals raises ROS production which causes oxidative stress and damage proteins, lipids, and nucleic acids (Gratão et al. 2015). Radish being important nutritive and economic vegetables is highly sensitive to several heavy metal stresses with no exception to cadmium. It is noticed that in certain localities of Pakistan cadmium concentration is significantly high, thus retarding the growth of plants and their yield. It is noteworthy to mention that cadmium can cause more damage to plants and human health than any other toxic heavy metal. Therefore, it is evident to found natural variation for Cd uptake and accumulation in root system of different radish genotypes which can be achieved through selection and screening of suitable radish germplasm with low-Cd-content.

Nanoparticles have been proved very useful for disease control and improving productivity of crop plants (Abdel-Aziz and Rizwan 2019). The supply of nanoparticles of iron (Fe) has been proved quite effective for plants but this positive effect depends on several factors i.e., crop species, concentration of nanoparticles, time of reaction of nanoparticles with plants (Hussain et al. 2019). Fe is considered a necessary component for physiological growth of any living organisms. It is also an important co-factor helping different enzymes for playing their role as catalyst in various biochemical processes (Briat et al. 2007). Nanoparticles of Fe have positive significanteffect on plant growth as compared to iron-based fertilizers (Elanchezhian et al. 2017). It has been proven that Fe nanoparticles triggered the growth and production of *Arachis hypogaea* and *Triticum aestivum* seedlings (Li et al., 2021). Similarly, various plant species damaged by cadmium toxicity can be cured by application of Fe_3_O_4_ nanoparticles (Hussain et al. 2019)

Some of the major benefits on application of Fe nanoparticles are improvement in growth, better transmission of material, and combination of new iron-oxide nanoparticles based fertilizers, may provide an alternate way to mitigate iron chlorosis and absorbing ions of heavy metals like cadmium (Sheykhbaglou et al. 2018). Therefore, the purpose of this study was to examine the monitoring role of Fe_3_O_4_ nanoparticles in radish forbearance under cadmium stress and to determine the adverse effect of cadmium heavy metal on studied radish cultivars. Furthermore, measuring growth, physiological and biochemical attributes of radish plants were recorded to analyze the effect of cadmium stress on radish plant which could be helpful for devising strategies to mitigate the negative impact of heavy metals on plant through application of nanoparticles.

## Materials and methods

### Seeds collection

The seeds of three cultivars of radish studied in present investigation were obtained from Ayub Agricultural Research Institute (AARI) Faisalabad, Pakistan (73º74 East, 30º31.5 North) during month of November (Table 1).

**Table 1:** Treatment and radish crop cultivars

No.	Treatments	Radish crop cultivars
1	Control	Mol Sano,
2	CdCl_2:_ 150 µM	Mol Hol Pari
3	Fe_3_O_4_:20 ppm	Mol Daq Wal
4	Fe_3_O_4_ 40 ppm	
5	Fe_3_O_4_:20 ppm + CdCl_2:_150 µM	
6	Fe_3_O_4_ 40 ppm + CdCl2: 150 µM	

### Workplace for experiment

The plants were grown in pots at experimental area of University of Education, Township Campus (N 31° 27’ 1.9224”, E 74° 17’ 58.398”) Lahore, Pakistan. Each pot was filled with 8 kg of air dried sandy loam soil with 7.2 pH, organic matter 0.90% and electrical conductivity 0.25mS/cm.

### Experimental layout

A sum of fifty-four pots were prepared to carry out this research. Among these, 18 pots were arranged for each cultivar and a completely randomized design was selected with three replicated to carry out this experiment. Three pots were treated with cadmium whereas, three were kept in control group and three were treated with 20 ppm of Fe_3_O_4_ whereas, three were treated with 40 ppm Fe_3_O_4_ nanoparticles. The last three pots were treated with cadmium and 40 ppm of Fe_3_O_4_ nanoparticles. The level of cadmium and Fe nanoparticles were selected based recommendations made by Rehman et al. (2018). However, effective concentration with little modification were selected for final application.

### Germination and thinning

The germination of seeds were completed in seven to eight days after sowing whereas, the growth of the seedlings started after five days of sowing. The thinning of seedlings were undertaken 14 days after germination and only 4 to 5 plants were kept in a pot.

### Stress application

14 days after germination the treatment of cadmium heavy metal was given through Hoagland solution and Fe_3_O_4_ was applied through foliar spray. After a month the treatment was repeated for the estimation of better results. Seven days after the second treatment data on physiological and biochemical parameters were recorded.

### Physiological and biochemical attributes

#### Determination of membrane permeability (%)

A sample of 0.5-gram plant was taken which was dipped for 24 h in 10 mL distilled water at room temperature. After 24 h dipped sample was vortex and prepared for EC reading later stored at 4^o^C. The sample was again vortexed after 24 h and recorded the next EC_1_ reading. After that, the sample was stored at 120 ^o^C for an hour which was later autoclaved for recordingreading EC_2_. The percentage relative permeability was calculated by using formulae given by Lutz et al. (2004).

$$\text{R}\text{M}\text{P} \left(\text{\%}\right)={\left[\frac{{EC}_{1}-{EC}_{0}}{{EC}_{2}-{EC}_{0}}\right]}_{ }\times 100$$

#### Determination of chlorophyll content

Method given by Arnon (1949) was followed to measure the chlorophyll contents in plants. We carefully weigh 0.5 g of fresh leaves sample along with 80% of acetone. The electronic balance was used to measure the weight of the first material, and later and mortar were used to grind the sample. The extract of the plant acquired after grinding was dissolved in 10 milliliter of acetone. The process of absorbance was studied with spectrophotometer at 480 nm, 645 nm and 663 nm wavelength.

Chlorophyll contents were determined by using following formula;

$$Chl.a (\text{m}\text{g}\,{ g}^{-1}\text{f}.\text{w}\text{t} )=\left[ 12.7 \left({OD}^{663}\right)-2.69 \left({OD}^{645}\right)\right\}\times \frac{V}{1000}\times W$$

$$\text{C}\text{h}\text{l}.\text{b}(\text{m}\text{g}\,{ g}^{-1}\text{f}.\text{w}\text{t} )=\left[ 22.9 \left( {OD}^{645}\right)-4.68 \left( {OD}^{663}\right)\right\}\times \frac{\text{V}}{1000}\times \text{W}$$

#### Determination of H_2_O_2_

Method given by Velikova et al. (1968) was used to analyze H_2_O_2_ in plant. We ground 0.5 g of sample through pestle and mortar and added 5 ml of 0.1% of trichloroacetic acid. The grinding procedure of this experiment was completed on ice bath. The material was centrifuged for making final sample and supernatant was taken for the analysis. We took 0.1 mL of phosphate buffer, added 2 mL KI solution (1 M) and 0.1 mL plant extract and run sample at spectrophotometer. The read absorbance of this mixture was taken at 390 nm of wavelength by using UV/VIS spectrophotometer.

#### Determination of malondialdehyde (MDA)

The MDA contents were determined by procedure given by Health and Pecker (1968). The 0.5 g leaf sample were dissolved in 100 milliliter of water to prepare thiobarbituric acid (TBA). The obtained 0.5 g of leaf extract were dissolved in 5 mL of TBA. The prepared mixture were kept at room temperature for 30 min. After 30 min, the mixture was heated for 15 min at 95 ^o^C in water bath, and immediately cooled the mixture on ice bath. The absorbance reading were recorded by using spectrophotometer at 450 nm, 523 nm and 600 nm of wavelength, respectively.

$$\text{MDA}\,\text{(nmol.}\,\text{Cm}^{-1}{)}\,=\,{1000}[\text{(Abs}_\text{523nm}-\text{Abs}_\text{600nm})/155]$$

#### Determination of anthocyanin

The ground sample of 0.5 g were mixed with 5 mL of phosphate buffer during grinding on ice bath. The centrifugation of sample was undertaken for homogenation. The supernatant was taken and kept for further analysis. By using spectrometer the absorbance of sample was measurd at 600 nm wavelength. The 2.65 g of KH_2_PO_4_ and 5.307 g of K_2_HPO_4_ were dissolved in 1 L of distilled water at pH 7 to prepare phosphate buffer.

#### Antioxidant enzyme activity

The ground 0.5 g of leaves sample was prepared on ice bath. The sample was dissolved into 5 mL of 50 mM phosphate buffer with 7.8 pH at the dissolved sample was centrifuged at 15,000 rpm for 20 min at 4^o^C temperature. Antioxidant enzyme activity was observed from the obtained supernatant.

#### Ascorbate peroxidase activity

Nkona and Asada (1981) method was followed to determine the ascorbate peroxide activity. According to this method 0.1 mL plant extract, 0.25 mM ascorbate, 0.1 mM H_2_O_2_, 0.1 mM EDTA and 25 mM sodium phosphate buffer were combined at pH 7.0. The enzyme oxidation of ascorbate was used to start enzyme activity and absorbance was determined at 290 nm (E = 2.8mM^− 1^cm^− 1^) wavelength.

#### Catalase and peroxidase

Chance and Maehly (1955) method was used to calculate the catalase and peroxidase. The 50 mM of phosphate buffer and 5.9 mM H_2_O_2_ were taken in test tube and pH 7.0 was maintained. The 0.1 mL plant extract was taken in test tube and reaction was undertaken by using enzyme extract. The reading of reaction solution was recorded at 240 nm after every 20 s. To determine the POD, 20 mM guaiacol, 50 mM phosphate buffer and 40 mM H_2_O_2_ were combined in a test tube. The 0.1 mL of enzyme extract was used to trigger the enzyme activity. The reading of solution was recorded at 70 nm wavelength after every 20 s. An absorbance at 0.01 units per minute was considered as POD activity.

#### Ion concentration

TO determine the ion concentration, sample was oven dried at 70^o^C. Furthermore, the concentrated sulphuric acid was used to dissolve the dried sample and kept for next 24 h. The dissolved flasks were heated at hot plate andH_2_O_2_ was added at regular intervals. The process of adding H_2_O_2_ continued until the mixture became colorless. In a 1000 mL beaker, 0.1 g coomassie brilliant blue were taken with 100 mL ortho-phosphoric acid, 50 mL of ethanol (95%) and 850 mL distilled water to prepare one liter of Bradford reagent.

#### Total soluble proteins

The o.5 gram of ground leaf sample was added into 50 mM phosphate buffer maintaining pH 7.8. The sample was centrifuged at6000 rotations per minutes for 20 min at 4^o^C temperature. The obtained supernatant was kept for further analysis and to measure the concentration of protein content in leaf extract the method of Bradford (1976) was followed. The 0.1 mL of leaf extract was mixed with 2 mL of Bradford reagent in a test tube. All test tubes were kept at room temperature for 5 min by using spectrophotometer and absorbance reading was recorded at 595 nm wavelength.

### Morphological aspects

The morphological parameters were measured i.e., fresh weight of shoots (g/ plant), fresh weight of roots (g /plant), root length (cm), shoot length (cm), dry weight of root (g/plant) and dry weight of shoot (g/plant) as per standard protocol.

### Statistical analysis

“Costat”, a statistical program, was employed to calculate analysis of variance for all studied parameters. Three factorial design with three completely randomized replications was employed to complete this procedure.

## Results

### Growth parameters

#### Root fresh weight (g)

**Fig. 1:**
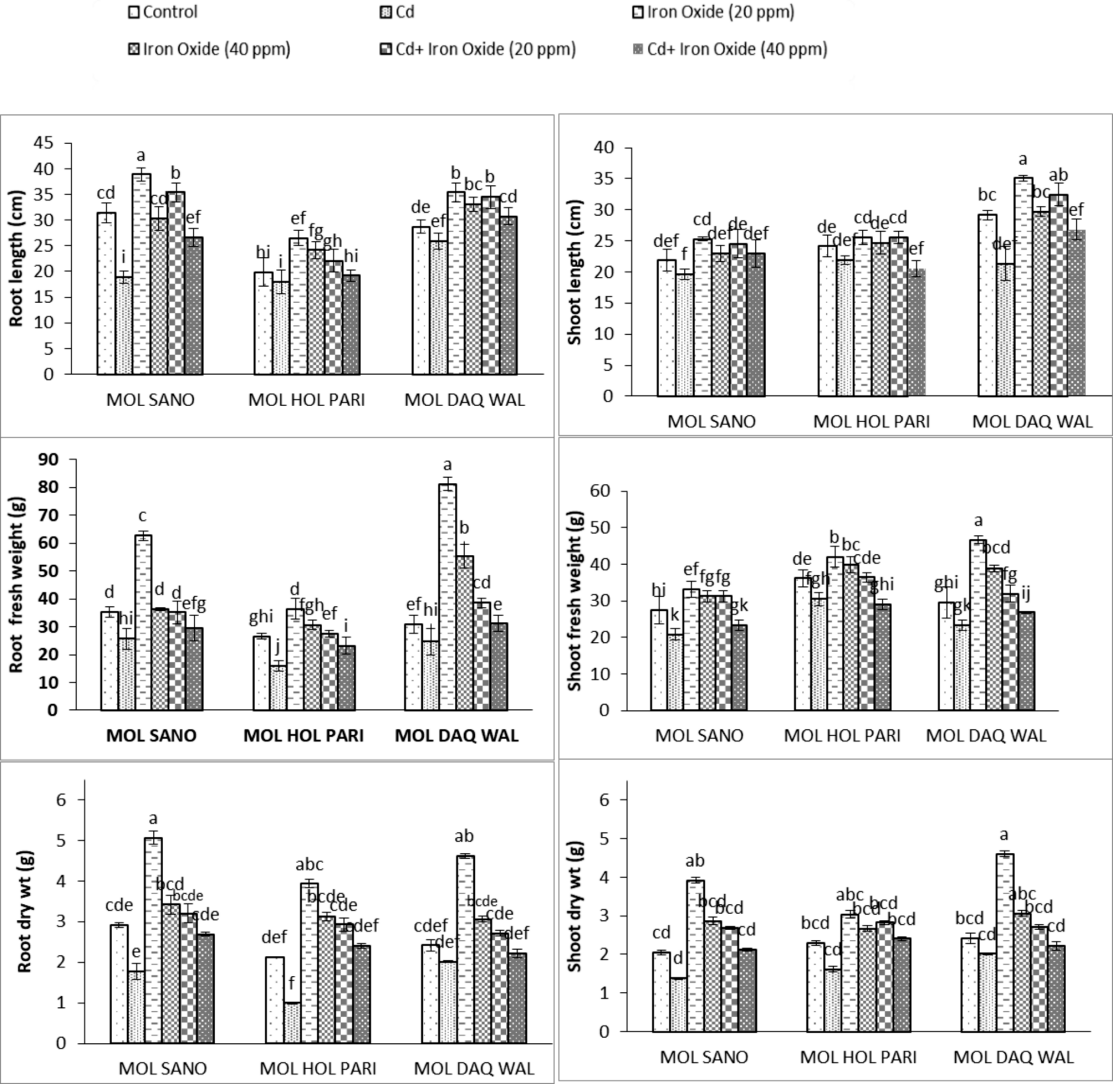
Root and Shoot fresh weight, dry weight and length of eight weeks old radish cultivars under cadmium and foliar spray of Fe_3_O_4_ nano-particles. ***(Bars of graph with identical letters in each group demonstrate that mean values are the same at 5% α)***

The foliar application of 20 mg/L Fe_3_O_4_ nanoparticles increased root fresh weight in all cultivars compared to 40 mg/L treatment of nanoparticles. The maximum values for root fresh weight were found in MOL-DAQ-WAL with treatment of 20 and 40 mg/L nanoparticles sprayed, respectively. Whereas, under combined treatment of cadmium and 20 mg/L Fe_3_O_4_ of nanoparticles root weight increase 0.75%, 3.29%,20.3% compared to control condition for all cultivars, respectively. The root weight shown reduction 19.8% and 14.7% in cultivars MOL- SANO and MOL-HOL-PARI oncecombined treatment of 40 mg/L and cadmium were given however, slightly (1.01%) increased in cultivar Mol-DAQ-Wal compared to control plants. Overall, highly significant interaction was found among all factors. The ANOVA showed that cadmium stress influence was variable in all varieties of radish (Fig. 1; Table 2).

**Table 2:** ANOVA of Root and Shoot fresh weight, dry weight and length of eight weeks old radish cultivars under and foliar spray of Fe_3_O_4_ nano particles

Source of variation	df	Root fresh weight	Root dry weight	Root length	Shoot fresh weight	Shoot dry weight	Shoot length
Cultivar	2	1916.8867***	1.600279***	513.92027 ***	280.8075***	0.7380***	200.00804 ***
Cadmium	1	3391.8208***	15.778817***	226.60712 ***	852.8363 ***	8.0736 ***	82.60934***
Fe_3_O_4_	2	1839.5169***	13.061569***	314.22334 ***	365.7665***	8.0986 ***	117.41007***
Variety * Cd	2	112.8449**	0.104517**	23.90367***	35.0572 **	0.5309 ***	13.47774***
Variety * Fe_3_O_4_	4	344.1411***	0.257452***	23.39609**	20.8879 **	0.2359 ***	15.66904***
Cd * Fe_3_O_4_	2	536.4707***	0.883206***	9.21381^ns^	20.0360*	0.3967***	10.09654*
Variety * Cd * Fe_3_O_4_	4	70.5104**	0.278872***	18.02212 **	18.6962**	0.3624***	5.91817^ns^

*Note* *, ** and *** significant at 0.05, df = degrees of freedom, ns = non-significant

#### Shoot fresh weight (g)

From ANOVA results it was found that shoot fresh weight declined remarkably once cadmium stress was applied in cultivars compared to control group. The shoot fresh weight for all cultivars were significantly different from each other (Table 2). The radish plants of all cultivars foliar sprayed with 20 mg/L of Fe_3_O_4_ nanoparticles showed that shoot fresh weight increased significantly compared to 40 mg/L foliar spray of Fe_3_O_4_ nanoparticles. The maximum increase in shoot fresh weight was recorded in cultivars MOL-DAQ-WAL followed by MOL-HOL-PARI and MOL SANO, respectively.

However, under combined cadmium treatment of 20 mg/L shoot fresh weight shown significant results for cultivars MOL-SANO. Whereas, under the combined treatment of 40 mg/L Fe_3_O_4_ nano-particles along with cadmium in comparison to control group shown 17.7362%, 24.652%, 9.89325% decrease in shoot fresh weight in all cultivars, respectively. Overall, significant interaction was found among all factors (Fig. 1).

#### Shoot length (cm)

The cadmium stress reduced the shoot length however, foliar application of 20 mg/L Fe_3_O_4_ increased the shoot length in all cultivars compared to control plants. Whereas, significant increase was observed in MOL- DAQ- WAL compared to other cultivars under investigation. The foliar treatment of 40 mg/L Fe_3_O_4_ was found non-significant for shoot length compared to control group. The foliar treatment of 20 mg/L Fe_3_O_4_ spray with cadmium increased shoot length from 10.93%, 5.49%, 10.118% for all cultivars, respectively. Moreover, the the combined treatment of 40 mg/L Fe_3_O_4_ nanoparticles and cadmium 4.98% in MOL SANO and 8.69% increase in MOL DAQ WAL values, whereas, 17.23% decrease in values of MOL- HOL- PARI were observed. Overall, non-significant interaction found among all factors. The ANOVA showed that in terms of shoot length results of all cultivars were significantly different (Fig. 1; Table 2).

#### **Root length (cm)**

The nanoparticles priming treatment of 20 mg/L Fe_3_O_4_ significantly elevated the root length in all investigated cultivars compared to 40 mg/L Fe_3_O_4_ nanoparticles treatment and maximum values were recorded for cultivar MOL SANO. Whereas, the treatment of 40 mg/L Fe_3_O_4_ nanoparticles t improved the root length in cultivars MOL HOL PARI and MOL DAQ WAL However, the cultivar MOL SANO displayed slight decrease in root length compared to control group. On contrary, a significant elevation of root length 11.24%, 10.3%, 16.71% was recorded under 20 mg/L of Fe_3_O_4_ nanoparticles of and cadmium for all cultivarscompared to control groupHowever, the combined treatment of cadmium and foliar application of 40 mg/L Fe_3_O_4_ nanoparticles shown reduction of17.74% in root length of cultivar MOL SANO followed by cultivar MOL HOL PARI were observed. Whereas, cultivar MOL DAQ WAL displayed 6.50% increase in values compared to control group under the combined interaction of cadmium and 40 mg/L of foliar applied Fe_3_O_4_ nanoparticles. Overall, the significant interaction was found among all factors studied. Furthermore, the ANOVA showed that significant decrease in root length under cadmium treatment in all cultivars (Fig. 1; Table 2).

#### Root dry weight (g)

ANOVA showed a highly significant difference among all cultivars (Fig. 1). A significant reduction for root dry weight was observed among all cultivars under cadmium stress. The foliar application of Fe_3_O_4_ nano-particles significantly increase root dry weight however, maximum values were recorded under 20 mg/L of Fe_3_O_4_ nanoparticles treatment followed by 40 mg/L Fe_3_O_4_ nanoparticles treatment compared to the control group. The combined application of cadmium and 20 mg/L Fe_3_O_4_ of nanoparticles increased 8.57%, 27.7%, 10.93% of root dry weight in all cultivars, respectively. On contrary, the combined treatment of 40 mg/L of Fe_3_O_4_ nanoparticles and cadmium shown reduction in root dry weight i.e., 8.56% for cultivar MOL SANO and 9.18% in MOL DAQ WAL cultivar however, MOL HOL PARI cultivar showed elevation of 11.9% in root dry weight. Overall, statistically significant interaction were found among all studied factors (Table 2).

#### Shoot dry weight (g)

Cadmium significantly reduced shoot dry weight in all cultivars under investigation however, Fe_3_O_4_ nanoparticles treatment elevated shoot dry weight compared to the control group. The maximum shoot dry weight were recorded under 20 mg/L of Fe_3_O_4_ nanoparticles followed by 40 mg/L Fe_3_O_4_ nanoparticles in cultivar MOL DAQ WAL. Under combined treatment of 20 mg/L of Fe_3_O_4_ nanoparticles and cadmium elevated the shoot dry weight in cultivar MOL SANO compared to control group. However, the combined application of 40 mg/L Fe_3_O_4_ of nanoparticles and cadmium shown 5.1% in cultivar MOL SANO and4.5% in MOL HOL PARI cultivar shoot dry weight. The cultivar MOL DAQ WAL shown9.7% of reduction for shoot dry weight. Overall significant interaction were found among all factors (Table 2). In terms of shoot dry weight, significant difference were observed forall three cultivars (Fig. 1).

### Physiological parameters

#### Membrane permeability

**Fig. 2:**
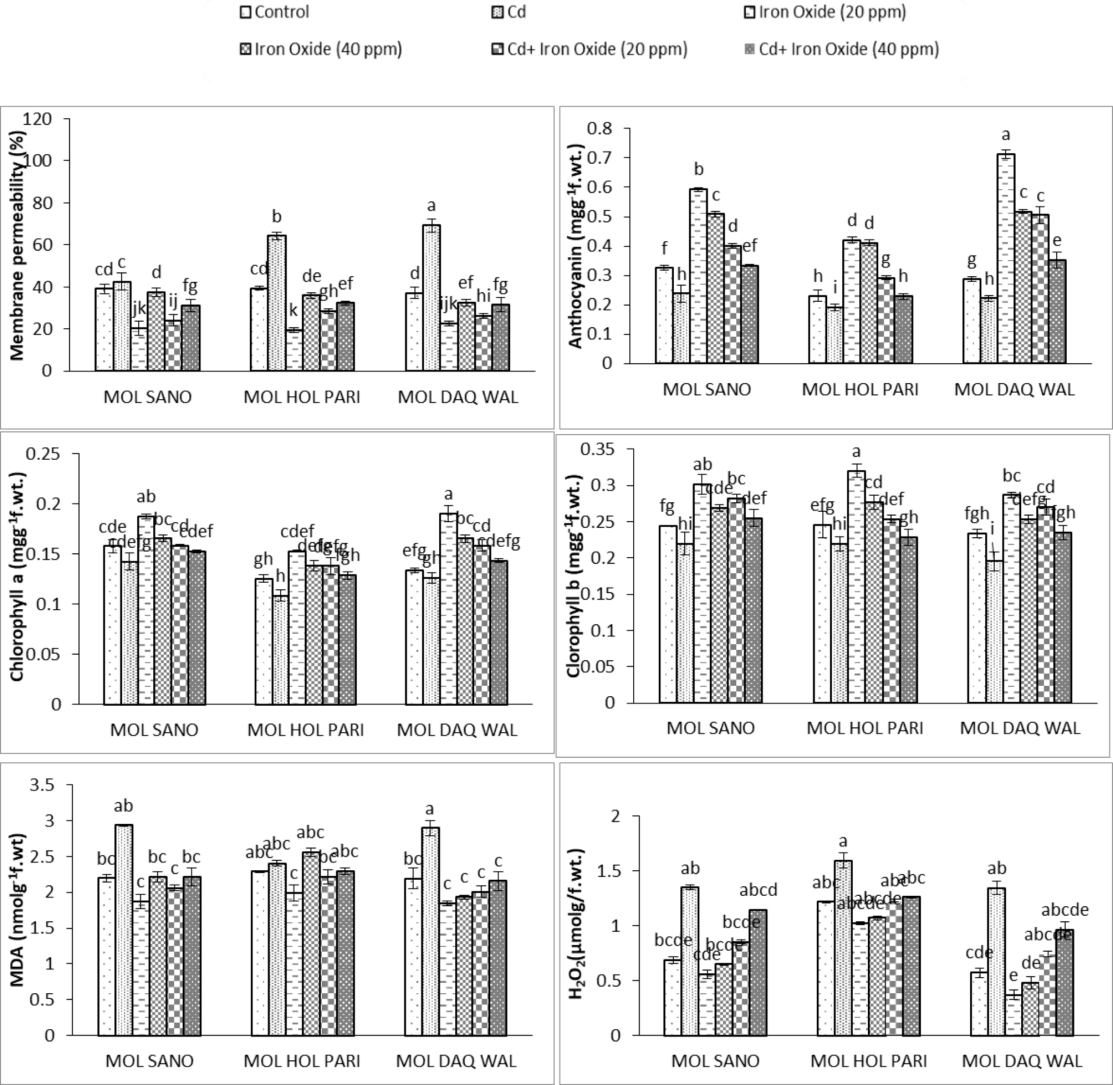
Membrane permeability, Anthocyanin, Chlorophyll a & b, MDA and Hydrogen peroxide (H_2_O_2_) content of eight weeks old radish under cadmium and foliar spray of Fe_3_O_4_ nano-particles. ***(Bars of graph with identical letters in each group demonstrate that mean values are the same at 5% α)***

Theimpact of Fe_3_O_4_ nanoparticles and cadmium stress on membrane permeability of all radish cultivars was significant. The membrane permeability of all cultivars increased under cadmium stress compared to the control group. Whereas, a significant reduction was detected in membrane permeability compared tocontrol group, under the higher dose of Fe_3_O_4_ nanoparticles. On contrary, the combined treatment of cadmium and foliar applied Fe_3_O_4_ nanoparticles shown significantly declined membrane permeability compared to the control plants. The maximum reduction was detected under 20 mg/L Fe_3_O_4_ nanoparticles treatment for cultivar MOL HOL PARI. Whereas, under 40 mg/L Fe_3_O_4_ nanoparticles treatment shown maximum reduction in membrane permeability for cultivar MOL DAQ WAL. Under the combined treatment of foliar applied 20 mg/L Fe_3_O_4_ and cadmium stress shown 62.2%, 38.3%, 41.9% reduction in membrane permeability compared to the control plants in all cultivars, respectively. On contrary, the combined treatment of cadmium and 40 mg/L Fe_3_O_4_ nanoparticles treatment shown 25.4%, 21.7%, 17.6% reduction for all cultivars under investigation compared tothe control group. Overall, statistically significance differences were observed among all factors (Table 3; Fig. 2).

**Table 3:** ANOVA of Membrane Permeability, Anthocyanin, Chlorophyll a + b, MDA and H_2_O_2_ content of eight weeks old radish cultivars under cadmium and foliar spray of Fe_3_O_4_ nano particles

Source of variation	df	Membrane Permeability	Anthocyanin	Chlorophyll a	Chlorophyll b	MDA	H_2_O_2_
Cultivar	2	103.6959***	0.091894***	0.0039637***	0.0012577***	0.063029***	1.14106***
Cadmium	1	723.1444***	0.256956***	0.0042715***	0.0124177***	0.732669***	2.49615***
Fe_3_O_4_	2	2871.5279***	0.258672***	0.0046204***	0.0158996 ***	1.074224***	0.49694***
Variety * Cd	2	167.5684***	0.001608**	0.0000546^ns^	0.0009672**	0.145563***	0.10144***
Variety * Fe_3_O_4_	4	95.1010***	0.017866 ***	0.0002062***	0.0000511^ns^	0.145429 ***	0.01496***
Cd * Fe_3_O_4_	2	649.9466***	0.018524***	0.0001915**	0.0000662^ns^	0.320291***	0.11216***
Variety * Cd * Fe_3_O_4_	4	96.2899 ***	0.001029**	0.0000832*	0.0004086**	0.064885***	0.00788***

*Note* *, ** and *** significant at 0.05, df = degrees of freedom, ns = non-significant

#### Anthocyanin

Cadmium stress significantly reduced anthocyanin in all radish cultivars compared to control condition and maximum reduction was observed in cultivar MOL HOL PARI. The application of 20 mg/L Fe_3_O_4_ nanoparticles significantly increased anthocyanin in cultivar MOL DAQ WAL under. However, the application of 40 mg/L Fe_3_O_4_ nanoparticles treatment significantly increased anthocyanin contents in cultivar MOL SANO. Moreover, the combined treatment of 20 mg/L Fe_3_O_4_ nanoparticles improved anthocyanin contents compared to control plants in all cultivars i.e., 18.81%, 20.9%, 41.11%, respectively. Whereas, combined treatment of 40 mg/L Fe_3_O_4_ nanoparticles and cadmium anthocyanin contents were improved in cultivar MOL SANO by 2.59% and 18.8% in cultivar MOL DAQ WAL compared to control condition whereas, 0.2% of anthocyanin contents were decreased in cultivar MOL HOL PARI ( compared tocontrol plants. The results of ANOVA displayed the significant difference under Fe_3_O_4_ nanoparticles and cadmium treatments among all cultivars (Table 3; Fig. 2).

#### Chlorophyll a

ANOVA results displayed that significance reductions was observed in chlorophyll a content for all cultivars of radish investigated in resent study. (Table 3). The treatment of 20 mg/L of Fe_3_O_4_ nanoparticles increased chlorophyll a were found in cultivar MOL DAQ WAL followed by foliar application of 40 mg/L Fe_3_O_4_ nanoparticles (Fig. 2). The Combined treatment of 20 mg/L of Fe_3_O_4_ nanoparticles and cadmium resulted elevation of 0.5%, 9.06%, 15.7% chlorophyll a contents compared to control condition in all cultivars, respectively. Whereas, under combined treatment of 40 mg/L, Fe_3_O_4_ nanoparticles and cadmium stress caused increase of 2.5% chlorophyll a in cultivar MOL HOL PARI and 6.09% in cultivar MOL DAQ WAL whereas, a slight reduction 3.03% of chlorophyll a content were observed in cultivar MOL SANO compared to control condition.

#### Chlorophyll b

Compared to chlorophyll a contents the chlorophyll b were significantly reduced under cadmium stress among all cultivars. The foliar treatment of Fe_3_O_4_ nanoparticles significantly improved chlorophyll a contents and maximum increase of chlorophyll a contents were observed in cultivar MOL HOL PARI under the treatment of 20 mg/L followed by treatment of 40 mg/L Fe_3_O_4_ nanoparticles. (Table 3; Fig. 2). The combined (20 mg/L, 40 mg/L) treatments of Fe_3_O_4_ nanoparticles and cadmium stress shown non-significant increase in chlorophyll b compared with the the control condition.

#### MDA content

The ANOVA results have shown that MDA contents of all cultivars significantly increased under cadmium stress compared to control plants. The treatment of 20 mg/L of MDA content significantly reduced compared to the control group and maximum reduction were detected in cultivar MOL DAQ WAL. Whereas, the treatment of 40 mg/L of Fe_3_O_4_ nanoparticles shown an elevation of 0.87% in cultivar MOL SANO and 10.4% in cultivar MOL HOL PARI. On contrary, the treatment of 40 mg/L of Fe3O4 nanoparticles in cultivar MOL DAQ WAL cultivar shown reduction of 14.08% for MDA contents compared to control group. Whereas, under combined treatment of 40 mg/L of Fe_3_O_4_ nanoparticles and cadmium 0.7% in cultivar MOL SANO and 0.3% in cultivar MOL HOL PARI increased MDA content compared to control group. Whereas, cultivar MOL DAQ WAL shown reduction of 1.5% in MDA content compared to control group. The combined treatment of 20 mg/L of Fe_3_O_4_ nanoparticles and cadmium shown reduction of 6.4%, 3.4%, 9.3%, respectively forMDA contents compared to control group for all three cultivars being investigated in present study (Table 3; Fig. 2).

#### Hydrogen peroxide content (H_2_O_2_)

The treatment of Fe_3_O_4_ nanoparticles caused reduction of H_2_O_2_ contents among all investigated cultivars. The treatment of 20 mg/L of Fe_3_O_4_ ameliorated stress significantly compared to treatment of 40 mg/L Fe_3_O_4_. The maximum reduction of in H_2_O_2_ contents was measured in cultivar MOL DAQ WAL at treatment of 20 mg/L Fe_3_O_4_ followed by 40 mg/L of Fe_3_O_4_ nano-particles. The combined treatment of 20 mg/L Fe_3_O_4_ nanoparticles and cadmium stress caused reduction of 5.95% in cultivar MOL SANO and 5.82% in cultivar MOL DAQ WAL for H_2_O_2_ contents compared to control group. in the cultivar MOL HOL PARI significantly increase of H_2_O_2_ contents compared to control group. The combined treatment of 40 mg/L of Fe_3_O_4_ nanoparticles and cadmium caused increase in H_2_O_2_ contents among all cultivars compared to control group. The ANOVA results displayed significant increase in H_2_O_2_ contents under cadmium stress for all radish cultivars (Table 3; Fig. 2).

### Inorganic ions

#### Sodium ion shoot (Na^+^)

**Fig. 3:**
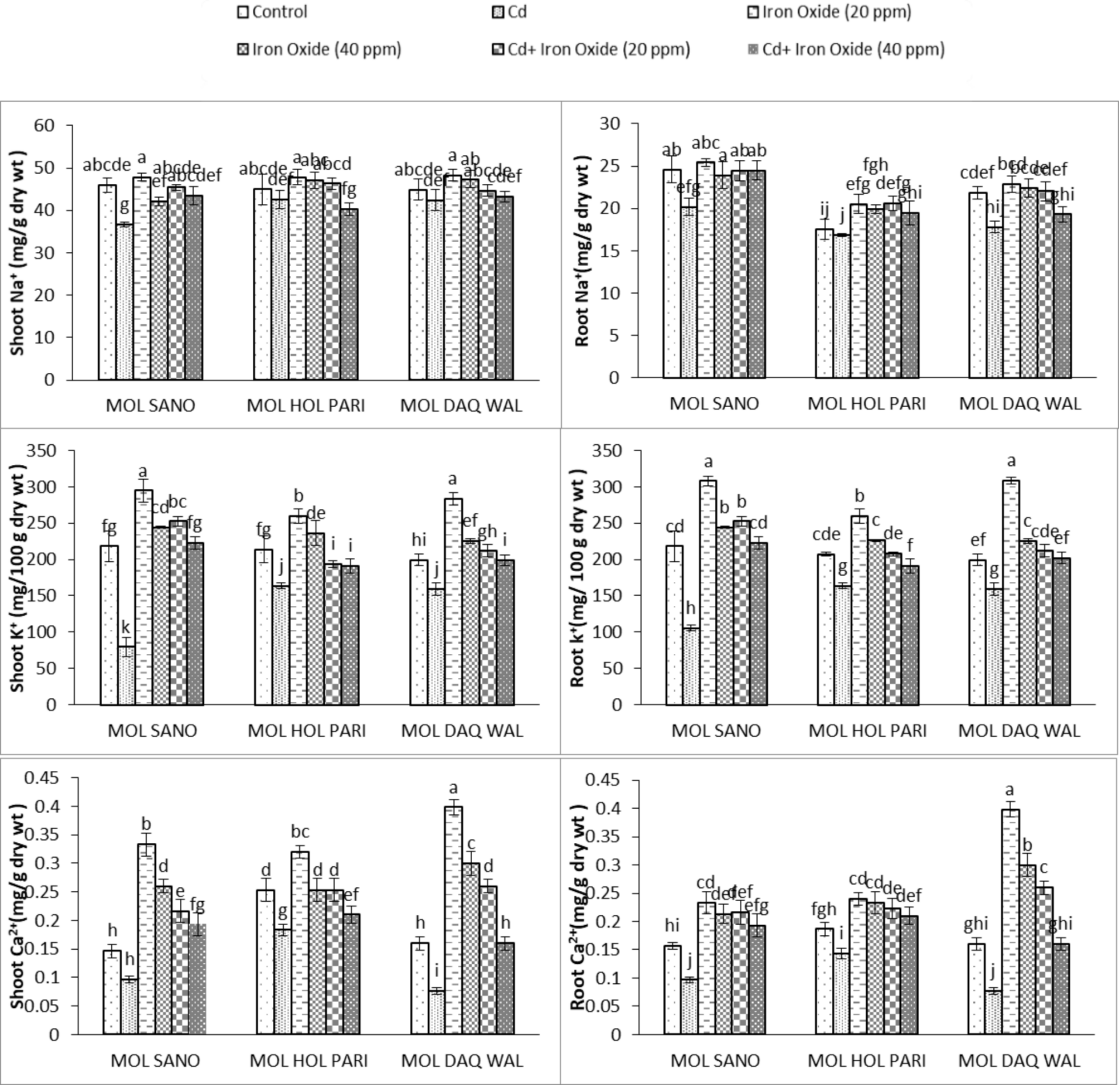
Root and Shoot Na, K and Ca content of eight weeks old radish under cadmium stress and foliar spray of magnetite Fe_3_O_4_ nano-particles. ***(Bars of graph with identical letters in each group demonstrate that mean values are the same at 5% α)***

The ANOVA results displayed a significant reduction in sodium in all cultivars of radish investigated in present study. The treatment of 20 mg/L OF Fe_3_O_4_ nano-particles displayed non-significant increase for sodium contents in all cultivars. However, the treatment of 40 mg/L of Fe_3_O_4_ nano-particles displayed 8.5% decrease in sodium content in cultivar MOL SANO whereas, an elevation of 4.39% in cultivar MOL HOL PARI and 5.2% in cultivar MOL DAQ WAL were observed. The combined treatment of 20 mg/L Fe_3_O_4_ nanoparticles and cadmium stress shown 1.1%, 2.8%, 0.4%, respectively reduction for sodium contents were observed for all cultivars, respectively. Similarily, the combined treatment of 40 mg/L Fe_3_O_4_ nano-particles and cadmium stress displayed 5.6%, 11.9%, 3.9% reduction in sodium contents were observed for all cultivars, respectively (Table 4; Fig. 3).

**Table 4:** ANOVA of Na, K and Ca of shoot and root of eight weeks old radish (*Raphanus sativus* L.) under cadmium and foliar spray of Fe_3_O_4_ nano particles

Source of variation	df	Shoot Na	Root Na	K shoot	Root K	Ca Shoot	Root Ca
Cultivar	2	12.68422*	100.12037***	348.42^ns^	1106.066***	0.0064241***	0.0076074***
Cadmium	1	162.20534***	31.77067***	42007.36***	38353.081***	0.1005352***	0.0492019***
Fe_3_O_4_	2	69.98637***	37.00544***	27146.13***	30568.743***	0.0940074***	0.0730796***
Variety * Cd	2	0.03618^ns^	5.93168**	534.46*	438.917 **	0.0044463**	0.0124741***
Variety * Fe_3_O_4_	4	4.47622^ns^	0.97742^ns^	3200.24***	2015.994***	0.0090046***	0.0089824***
Cd * Fe_3_O_4_	2	5.86206^ns^	8.01975**	2308.56***	2369.855***	0.0018296**	0.0000407^ns^
Variety * Cd * Fe_3_O_4_	4	25.42130***	3.07064*	2251.36 ***	1557.577***	0.0009491**	0.0013713***

*Note* *, ** and *** significant at 0.05, df = degrees of freedom, ns = non-significant

#### Sodium ion root (Na^+^)

The ANOVA results revealed that cadmium stress has reduced the root’s sodium significantly. The treatment of 20 mg/L of Fe_3_O_4_ treatment elevated 3.15%, 14.43%, 4.37% sodium content in all radish cultivars, respectively. Whereas, the treatment of 40 mg/L of Fe_3_O_4_ increased 2.86% of sodium ion content in cultivar MOL SANO and 11.6% in cultivar MOL HOL PARI whereas reduced 2.48% in cultivar MOL DAQ. The combined treatment of 20 mg/L of Fe_3_O_4_ nanoparticles and cadmium stress caused 0.8%, 14.8%, 0.8% increase in sodium ion content in all cultivars, respectively. Whereas, the combined treatment of 40 mg/L of Fe_3_O_4_ nano-particles and cadmium stress decreased 0.5%, 9.3%, 13.3% in sodium ions in all cultivars, respectively (Fig. 3).

#### Potassium ion shoot (K^+^)

The application of 20 mg/L Fe_3_O_4_ nano-particles increased shoot potassium values compared to the treatment of 40 mg/L Fe_3_O_4_ of nanoparticles. The maximum values of shoot potassium was observed under 20 mg/L Fe_3_O_4_ treatment followed by 40 mg/L Fe_3_O_4_ treatment in cultivar MOL DAQ WAL compared to the control plants. The combined treatment of 20 mg/L Fe_3_O_4_ nanoparticles and cadmium stress shown elevation of 13.6%, 10.2%, 5.8% of potassium ion for all cultivars compared to the control group. Whereas, the treatment of 40 mg/L of Fe_3_O_4_ nanoparticles and cadmium increased 1.91% in potassium ion concentration in cultivar MOL SANO whereas, 12.02% increase was observed in cultivar MOL HOL PARI and 0.2% reduction was observed in cultivar MOL DAQ WAL compared tothe control group. The ANOVA results revealed that significant variations were observed in all cultivars of radish plant andcadmium stress significantly decreases potassium of shoot in all cultivars (Table 4; Fig. 3).

#### Potassium ion root (K^+^)

The treatment of 20 mg/L Fe_3_O_4_ of nanoparticles priming significantly increased root potassium compared to 40 mg/L of Fe_3_O_4_ nanoparticles treatment in all cultivars under investigation. The maximum values of root potassium ion were observed in cultivar MOL DAQ WAL under 20 mg/L followed by 40 mg/L of Fe_3_O_4_ nanoparticles treatment. However, the combined treatment of 20 mg/L of Fe_3_O_4_ nanoparticles and cadmium caused13.6%, 0.4%, 5.8%, respectively increase in root potassium ions compared to the control plants in all cultivars. Whereas, the combined treatment of 40 mg/L of Fe_3_O_4_ nanoparticles and cadmium caused an increase of 1.9% in cultivar MOL SANO and 1.4% in cultivar MOL DAQ WAL, respectively for root potassium ions whereas, cultivar MOL HOL PARI shown reduction of 8.5% in root potassium ions compared to the control group (Table 4; Fig. 3).

#### Calcium ion shoot (Ca^+^)

The ANOVA results displayed a significant difference for calcium ions in shoots of cultivars being investigated in present study. The application of cadmium significantly reduced the calcium ions in shoots of all cultivars of radish. The Fe_3_O_4_ nanoparticles priming increased calcium ions of shoot and maximum increase was observed in calcium ions in cultivar MOL DAQ WAL with a treatment of 20 mg/L of Fe_3_O_4_ nanoparticles followed by 40 mg/L of Fe_3_O_4_ nanoparticles compared to control plants. The combined treatment of 20 mg/L Fe_3_O_4_ of nanoparticles caused 32.3% and 38.4% increase in calcium ions of shoot compared to the control plants in cultivar MOL SANO and MOL DAQ WAL, respectively whereas, no significant increase was observed in calcium ions of shoot in cultivar MOL HOL PARI compared to the control plants. The combined treatment of 40 mg/L of Fe_3_O_4_ nanoparticles and cadmium shown an increase of shoot calcium ions compared to the control plants in cultivar MOL SANO. (Table 4; Fig. 3).

#### Calcium ion root (Ca^+^)

The ANOVA results displayed that application of cadmium caused highly significant reduction of calcium in roots compared to control group of radish cultivar being investigated in present study (Table 4). The treatment of 20 mg/L of Fe_3_O_4_ nanoparticles elevated 37.1%, 22.2%, 59.8%, respectivelyof root calcium compared to control plants. Whereas, the treatment of 40 mg/L of Fe_3_O_4_ cuased an increase of32.2% for cultivar MOL SANO and 46% in cultivar MOL DAQ WAL, respectively in root calcium compared to the control plants and. Whereas, the cultivar MOL HOL PARI shown no difference for root calcium compared to the control group. The treatment 20 mg/L Fe_3_O_4_ nanoparticle s and cadmium caused an elevation of 32.32%, 16.24%, 38.47%, respectively for root calcium in all cultivars compared to control group. The foliar application of 40 mg/L of Fe_3_O_4_ nanoparticles and cadmium displayed 24.4% increase in root calcium in cultivarMOL SANO cultivar 24.4% compared to the control group, whereas, the other two cultivars shown no variation for root calcium contents (Fig. 3).

### Biochemical attributes

#### Proteins

**Fig. 4:**
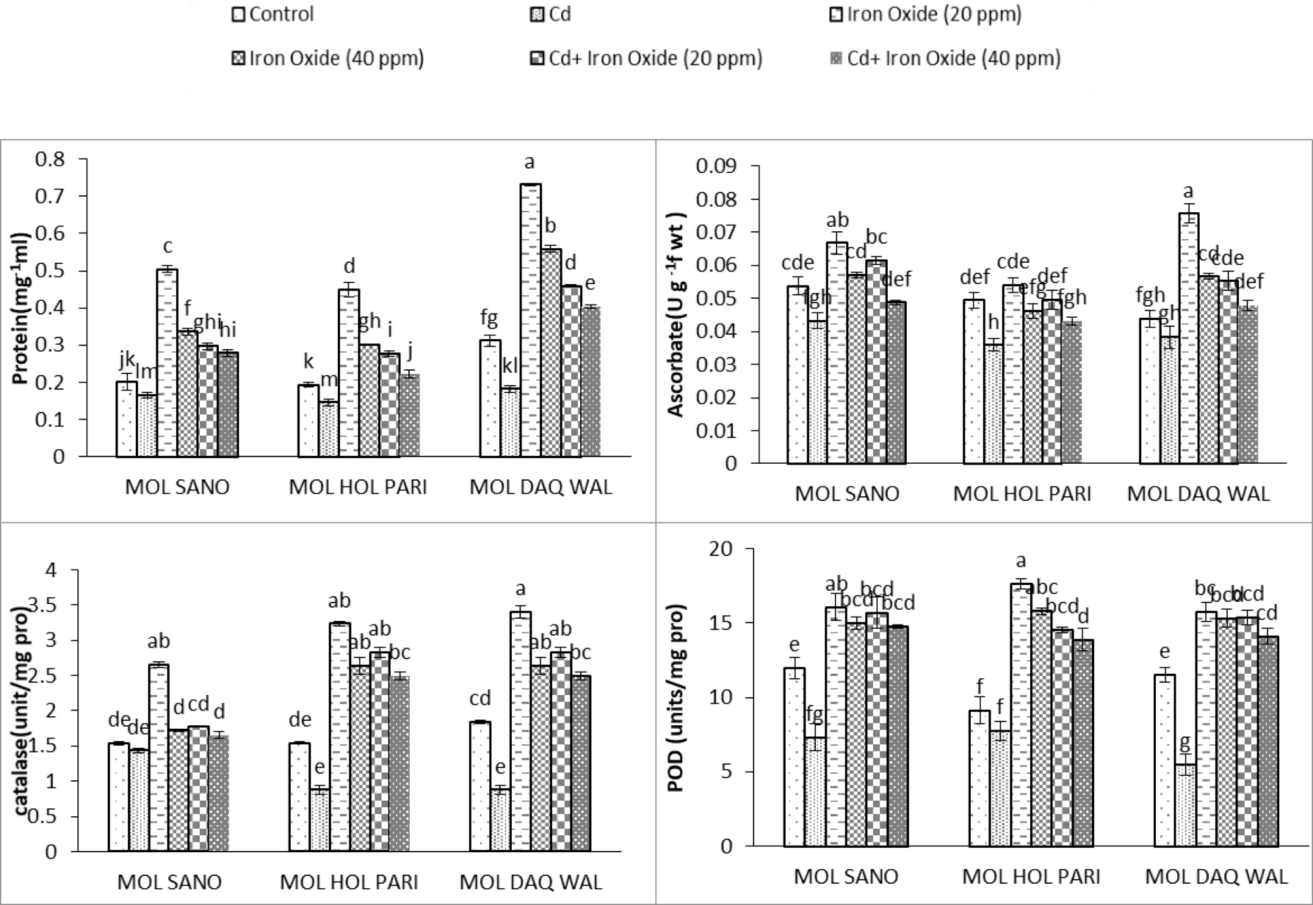
Protein, Ascorbate peroxidase, Catalase and peroxidase of eight weeks old radish cultivars under cadmium and foliar spray of magnetite Fe_3_O_4_ nano-particles. ***(Bars of graph with identical letters in each group demonstrate that mean values are the same at 5% α)***

The ANOVA results displayed statistically significant difference for protein among all cultivars. The results displayed that cadmium treatment significantly reduced protein content among all radish cultivars. The foliar 20 mg/L of Fe_3_O_4_ nanoparticles increased protein content among all cultivars under compared to the treatment of 40 mg/L of Fe_3_O_4_ nanoparticles. The significant increase in protein content were noted in cultivar MOL DAQ WAL under treatment of 20 mg/L of Fe_3_O_4_ nanoparticles followed by treatment of 40 mg/L Fe_3_O_4_ nanoparticles. The combined treatment of 20 mg/L of Fe_3_O_4_ nanoparticles and cadmium increased30.7%, 30.2%, 32.1%, respectively of protein content compared to control group of all cultivars. Whereas, the combined treatment of 40 mg/L of Fe_3_O_4_ nanoparticles and cadmium shown elevation of 28.1%, 13.2%, 22.6%, respectively in protein content as compared to control plants of all cultivars (Table 5; Fig. 4).

**Table 5:** ANOVA of Protein, Ascorbate, Catalase and Peroxidase of eight weeks old radish cultivars under cadmium and foliar spray of magnetite Fe_3_O_4_ nano particles

Source of variation	Df	Protein	Ascorbate	Catalase	POD
Cultivar	2	0.15807***	0.003763***	5.62462***	60.59741***
Cadmium	1	0.22238***	0.001062***	10.97103***	302.12876***
Fe_3_O_4_	2	0.29241***	0.001237***	10.34871***	257.06002***
Variety * Cd	2	0.01119***	0.000253*	0.08075***	11.12120***
Variety * Fe_3_O_4_	4	0.01256***	0.000899***	0.04581 ***	12.89131***
Cd * Fe_3_O_4_	2	0.02728***	0.000157^ns^	3.52485***	11.26242***
Variety * Cd * Fe_3_O_4_	4	0.00034*	0.000677**	0.42846 ***	4.37085***

*Note* *, ** and *** significant at 0.05, df = degrees of freedom, ns = non-significant

#### Ascorbate peroxidase

A significant elevation for ascorbate peroxidase were observed at treatment of 20 mg/L of Fe_3_O_4_ nanoparticles followed by 40 mg/L of Fe_3_O_4_ nanoparticles treatment in cultivar MOL DAQ WAL. The combined treatment of 20 mg/L of Fe_3_O_4_ nanoparticles and cadmium shown an elevation of 12% in cultivar MOL SANO and 20.6% in cultivar MOL DAQ WAL ascorbate peroxidase compared to control plants. in the cultivar MOL HOL PARI shown slight reduction of ascorbate peroxidase compared to control plants. Whereas, the combined treatment of 40 mg/L of Fe_3_O_4_ nanoparticles and cadmium shown reduction of 10.25% in cultivar MOL SANO and 14.8% in cultivar MOL HOL PARI for ascorbate peroxidase whereas, the cultivar MOL DAQ WAL shown an elevation of 8.4% for ascorbate peroxidase compared to the control plants. The ANOVA results showed highly significant variation for ascorbate peroxidase amongall cultivars. Moreover, it was observed that cadmium stress shown significant reduction of ascorbate peroxidase enzyme activity among all cultivars (Fig. 4; Table 5).

#### Catalase

The CAT activity was decreased significantly under Fe_3_O_4_ nanoparticles treatment in all radish cultivars, but cadmium treatment caused elevation for catalase. However, catalase values for recorded lowest in cultivar MOL DAQ WAL under 20 mg/L of Fe_3_O_4_ nanoparticles followed by 40 mg/L of Fe_3_O_4_ nanoparticles treatment compared to the control group. The combined treatment of 20 mg/L of Fe_3_O_4_ nanoparticles and cadmium caused a reduction of 8.7%, 6.9%, 22%, respectively forCAT activity in all cultivars. Whereas, the combined treatment of 40 mg/L of Fe_3_O_4_ nanoparticles and cadmium shown a reduction of 6.2%, 6.9%, 9.1% for CAT activity compared to control plants of all cultivars, respectively (Table 5; Fig. 4).

#### Peroxidase (POD)

The ANOVA results displayed the activity of peroxidase enzyme increased significantly in all radish cultivars investigated in present study. The minimum values were recorded for peroxidase enzyme in cultivar MOL SANO at treatment of 20 mg/L of Fe_3_O_4_ nanoparticles followed by 40 mg/L of Fe_3_O_4_ nanoparticles treatment. The combined treatment of 20 mg/L of Fe_3_O_4_ nanoparticles and cadmium showed reduction of 37%, 4.01% and 7.7% of peroxidase enzyme activity compared to the control group for all cultivars, respectively. The combined treatment of 40 mg/L of Fe_3_O_4_ nanoparticles and cadmium showed a reduction of 5.7%, 0.3%, 1.5% for peroxidase enzyme compared to the control group, respectively (Table 5; Fig. 4).

## Discussion

The heavy metal contamination particularly cadmium is among hotspot among researchers and considered highly destructive stress for both plants and human health. Cadmium cause several invisible damages to plants such as necrosis, chlorosis etc. The contamination may alter the plant growth processes like metabolism, mineral nutrition and transpiration etc. (Gill et al. 2013). To minimize toxicity of cadmium, plants have developed mechanisms i.e., exclusion by sugar and alcohols. The use of iron based nano-fertilizers mayprovide an alternate solution to eliminate the iron chlorosis symptoms, enhance growth and nutritional quality (Sheykhbaglou et al. 2018; Shakoor et al. 2022). Some studies have revealed that metals and their respective oxides of nanomaterial are dangerous for plant health whereas, some studies have shown that these particles are beneficial for the plant growth and productivity (Cvjetko et al. 2017; Okupnik and Pflugmacher 2016; Tripathi et al. 2017). The nanoparticles of Fe_3_O_4_ are considered effective, for plant health and growth once applied at specific concentration. The magnetite Fe_3_O_4_ is vital for plant’s physiological processes, protein contents and photosynthesis (Wang et al. 2015). It also have quality to bear environmental stresses and to scavenge oxygen radicals (Konate et al. 2017).

In this present investigation the mitigating role of Fe_3_O_4_ nanoparticles on cadmium stress was studied. The mitigation effectiveness of magnetite Fe_3_O_4_ nanoparticles on the bases of their impact on growth, biochemical, antioxidant enzymatic potential. The cadmium treatment reduced shoot fresh weight, root fresh weight, length of root and shoot, dry weight of root and shoot of all radish cultivars was significant (Fig. 1; Table 2). It is also reported that an increase in concentration of cadmium decreases fresh weights of shoot and root of radish plant significantly (Pirdosti et al. 2019). Moreover, this treatment highly significantly impacted biochemical parameters i.e., anthocyanin, chlorophyll a, chlorophyll b contents, soluble proteins got diminished under cadmium stress. El-Beltagi et al., (2010) also reported the similar results that increasing cadmium concentration decreased chlorophyll a, b contents causing membrane damage by increasing membrane permeability under cadmium stress (Fig. 2; Table 3). The activity of CAT, POD and antioxidants profiles got significant elevation under cadmium stress among in all radish cultivars being studied in present studies. Xu et al. (2020) reported similar results under 150 µM cadmium stress, El-Beltagi et al. (2010) also reported comparable results. The significant increase in H_2_O_2_ and MDA content were observed in all radish cultivars compared to the control group and Amirabad et al. (2020) also presented similar results.

The treatments of radish with Fe_3_O_4_ nanoparticles improved morphological, physiological, and biochemical attributes significantly in all radish cultivars compared to the control group. Wang et al. (2015) and Li et al. (2021) also reported similar results under Fe_3_O_4_ nanoparticles treatment. The 20 mg/L Fe_3_O_4_ nanoparticles improvement in morphological, physiological and biochemical attributes were more significant compared to the 40 mg/L magnetic Fe_3_O_4_ nanoparticles. On contrary Zadeh et al. (2019) also determined that magnetic Fe_3_O_4_ nanoparticles could nullify cadmium stress and improved the plant growth, ions concentrations, chlorophyll contents and antioxidants enzymatic activities. Whereas, non-significant increase in Na ions concentrations were found under Fe_3_O_4_ nanoparticles treatment in comparison with control group, the similar results were reported by Askary et al. (2016).

## Conclusion

The present work concluded that magnetic Fe_3_O_4_ nanoparticles treatment can induce tolerance in *Raphanus sativus L*. against cadmium stress. The cadmium stress causes significant reduction in morphological, physiological, biochemical attributes i.e., root length, root fresh weight, shoot fresh weight, length, chlorophyll (a and b) content anthocyanin, ions concentrations, proteins content, APX activity while, membrane permeability, H_2_O_2,_ POD, MDA, and CAT activity got increased. However, Fe_3_O_4_ nanoparticles foliar spray treatments mitigated cadmium stress toxic effects on all radish cultivars. Whereas, 20 mg/L Fe_3_O_4_ nanoparticles have been proven more useful in enhancing all study parameters in *Raphanus sativus L*.

## Data Availability

All relevant data is present within the article and supplementary files.

## References

[CR1] Abdel-Aziz HMM, Rizwan M (2019). Chemically synthesized silver nanoparticles induced physio-chemical and chloroplast ultrastructural changes in broad bean seedlings. Chemosphere.

[CR2] Amal AM, Hossam SEB, Mohamed MR (2009). Cadmium stress induced change in some hydrolytic enzymes, free radical formation and ultrastructural disorders in radish plant. Electron J Environ Agricultural Food Chem.

[CR3] Arnon DI (1949). Copper enzymes in isolated chloroplasts. Polyphenoloxidase in Beta Vulgaris. Plant Physiol.

[CR4] Asgher M, Khan MIR, Anjum NA, Khan NA (2015). Minimising toxicity of cadmium in plants—role of plant growth regulators. Protoplasma.

[CR5] Askary M, Talebi SM, Amini F, Bangan ADB (2016). Effect of NaCl and Fe_3_O_4_ nanoparticles on Mentha Piperita essential oil composition. Environ Experimental Biology.

[CR6] Auobi Amirabad S, Behtash F, Vafaee Y (2020). Selenium mitigates cadmium toxicity by preventing oxidative stress and enhancing photosynthesis and micronutrient availability on radish (Raphanus sativus L.) cv. Cherry Belle. Environ Sci Pollut Res.

[CR7] Bombin S, LeFebvre M, Sherwood J, Xu Y, Bao Y, Ramonell KM (2015). Developmental and reproductive effects of Fe_3_O_4_ nanoparticles in arabidopsis thaliana. Int J Mol Sci.

[CR8] Briat J-F, Curie C, Gaymard F (2007). Iron utilization and metabolism in plants. Curr Opin Plant Biol.

[CR9] Chance B, Maehly AC (1955). Assayofcatalasesandperoxidases Methods Enzymol.

[CR10] Cvjetko P, Milosic A, Domijan A-M, Tkalec M, Balen B (2017). Toxicity of silver ions and differently coated silver nanoparticles in Allium cepa roots. Ecotoxicol Environ Saf.

[CR11] EL-Beltagi HS, Mohamed AA, Rashed MM (2010). Response of Antioxidative Enzymes to cadmium stress in leaves and roots of Radish (Raphanus sativus L). Notulae Scientia Biologicae.

[CR12] Elanchezhian R, Kumar D, Ramesh K, Biswas AK, Guhey A, Patra AK (2017). Morpho-physiological and biochemical response of maize (Zea mays L.) plants fertilized with nano-iron (Fe3O4) micronutrient. J Plant Nutr.

[CR13] Fahad, Balouch A, Agheem MH, Memon SA, Baloch AR, Tunio A, Abdullah, Pato AH, Jagirani MS, Panah P, Gabole AA, Qasim S (2020). Efficient mitigation of cadmium and lead toxicity in coriander plant utilizing magnetite (Fe3O4) nanofertilizer as growth regulator and antimicrobial agent. Int J Environ Anal Chem.

[CR14] Gill SS, Hasanuzzaman M, Nahar K, Macovei A, Tuteja N (2013). Importance of nitric oxide in cadmium stress tolerance in crop plants. Plant Physiol Biochem.

[CR15] Gratão PL, Monteiro CC, Tezotto T, Carvalho RF, Alves LR, Peters LP, Azevedo RA (2015). Cadmium stress antioxidant responses and root-to-shoot communication in grafted tomato plants. Biometals.

[CR16] Hasan SA, Hayat S, Ahmad A (2011). Brassinosteroids protect photosynthetic machinery against the cadmium induced oxidative stress in two tomato cultivars. Chemosphere.

[CR17] Health RL, Pecker L (1968). Photoperoxidation in isolated chloroplasts: I. kinetics and stoichiometry of fatty acid peroxidation. Arch Biochem Biophys.

[CR18] Hussain F, Hadi F, Akbar F (2019). Magnesium oxide nanoparticles and thidiazuron enhance lead phytoaccumulation and antioxidative response in Raphanus sativus L. Environ Sci Pollut Res.

[CR19] Jalali M, Ghanati F, Modarres-Sanavi AM (2016). Effect of Fe_3_O_4_ nanoparticles and iron chelate on the antioxidant capacity and nutritional value of soil-cultivated maize (Zea mays) plants. Crop Pasture Sci.

[CR20] Kao CH (2014). Cadmium stress in rice plants: influence of essential elements. Crop Environ Bioinform.

[CR21] Konate A, He X, Zhang Z, Ma Y, Zhang P, Alugongo GM, Rui Y (2017). Magnetic (Fe3O4) nanoparticles reduce heavy metals uptake and mitigate their toxicity in wheat seedling. Sustainability.

[CR22] Kopta T, Pokluda R (2013). Yields, quality and nutritional parameters of radish (Raphanus sativus) cultivars when grown organically in the Czech Republic. Hortic Sci.

[CR23] Li M, Zhang P, Adeel M, Guo Z, Chetwynd AJ, Ma C, Rui Y (2021). Physiological impacts of zero valent iron, Fe_3_O_4_ and Fe_2_O_3_ nanoparticles in rice plants and their potential as fe fertilizers. Environ Pollut.

[CR24] Liu Y, Yu X, Feng Y, Zhang C, Wang C, Zeng J, Huang Z, Kang H, Fan X, Sha L, Zhang H, Zhou Y, Gao S, Chen Q (2017). Physiological and transcriptome response to cadmium in cosmos (Cosmos bipinnatus Cav.) Seedlings. Sci Rep.

[CR25] Lutz A, Greischar LL, Rawlings NB, Ricard M, Davidson RJ (2004). Gamma Synchrony Dur Mental Pract Pnas.

[CR26] Okupnik A, Pflugmacher S (2016). Oxidative stress response of the aquatic macrophyte Hydrilla verticillata exposed to TiO2 nanoparticles. Environ Toxicol Chem.

[CR27] Pirdosti KM, Movahedi Z, Rostami M (2019). Effect of cadmium stress on morpho-physiological traits in garden cress and radish in an aeroponic system. Iran J Plant Physiol.

[CR30] Rui M, Ma C, Hao Y, Guo J, Rui Y, Tang X, Zhao Q, Fan X, Zhang Z, Hou T, Zhu S (2016). Fe_3_O_4_ nanoparticles as a potential iron fertilizer for peanut (Arachis hypogaea). Front Plant Sci.

[CR31] Shakoor N, Adeel M, Zain M, Zhang P, Ahmad MA, Farooq T, Rui Y (2022). Exposure of cherry radish (Raphanus sativus L. var. Radculus Pers) to iron-based nanoparticles enhances its nutritional quality by trigging the essential elements. NanoImpact.

[CR32] Sheykhbaglou R, Sedghi M, Fathi-Achachlouie B (2018) The Effect of Ferrous Nano-oxide particles on physiological traits and nutritional compounds of soybean (Glycine max L.) seed the Effect of Ferrous Nano-oxide particles on physiological traits and nutritional compounds of soybean (Glycine max L.) seed. An Acad Bras Cienc 89(4). 10.1590/0001-3765201820160251 10.1590/0001-3765201820160251 29466478

[CR33] Tripathi DK, Singh S, Singh S, Shrivastav PK, Singh PK, Singh VP (2017). Nitric oxide alleviates silver nanoparticles (AgNps)-induced phytotoxicity in Pisum sativum seedlings. Plant Physiol Biochem.

[CR34] Varalakshmi LR, Ganeshamurthy AN (2013). Phytotoxicity of Cadmium in Radish and its effects on Growth, Yield, and Cadmium Uptake. Commun Soil Sci Plant Anal.

[CR35] Velikova V, Yordanov I, Edreva A (1968). Interference, mixing, and angular correlations in decays of boson resonances. Phys Rev.

[CR36] Vitória AP, Cunha D, Azevedo RA (2006). Ultrastructural changes of radish leaf exposed to cadmium. Environ Exp Bot.

[CR37] Wang M, Liu X, Hu J, Li J, Huang J (2015). Nano-Ferric Oxide promotes Watermelon Growth. J Biomaterials Nanobiotechnol.

[CR101] Xu L, Zhang F, Tang M, Wang Y, Dong J, Ying J, Chen Y, Hu B, Li C, Liu L (2020) Melatonin confers cadmium tolerance by modulating critical heavy metal chelators and transporters in radish plants. J Pineal Res 69(1):e12659 10.1111/jpi.12659 32323337

[CR38] Zadeh RR, Arvin SMJ, Jamei R, Mozaffari H, Reza Nejhad F (2019). Response of tomato plants to interaction effects of magnetic (Fe3O4) nanoparticles and cadmium stress. J Plant Interact.

[CR39] Zaki MM, El-Midany SA, Shaheen HM, Rizzi L (2012). Mycotoxins in animals: occurrence, effects, prevention and management. J Toxicol Environ Health Sci.

[CR28] Zia-ur-Rehman M, Naeem A, Khalid H, Rizwan M, Ali S, Azhar M (2018) Responses of plants to iron oxide nanoparticles. In Durgesh Kumar Tripathi, Parvaiz Ahmad, Shivesh Sharma, Devendra Kumar Chauhan, Nawal Kishore Dubey (eds), Nanomaterials in Plants, Algae, and Microorganisms, Academic Press (2018), pp 221–238, ISBN 9780128114872

